# Feasibility and efficacy of salvage allogeneic stem cell transplantation in AML patients relapsing after autologous stem cell transplantation

**DOI:** 10.1038/s41409-021-01521-5

**Published:** 2021-11-13

**Authors:** Evgenii Shumilov, Inna Shakhanova, Johanna Flach, Nicole Schmidt, Susanne Buerki, Myriam Legros, Marie-Noëlle Kronig, Yishai Ofran, Sabine Gerull, Michael Medinger, Behrouz Mansouri Taleghani, Jakob Passweg, Jörg Halter, Ulrike Bacher, Thomas Pabst

**Affiliations:** 1 Department of Medical Oncology, Inselspital, Bern University Hospital, Bern, Switzerland; 2 Department of Hematology and Medical Oncology, University Medicine Göttingen (UMG), Göttingen, Germany; 3 Department of Nephrology and Rheumatology, University Medicine Göttingen (UMG), Göttingen, Germany; 4 Department of Hematology and Central Hematology Laboratory, Inselspital, Bern University Hospital, Bern, Switzerland; 5 Center of Laboratory Medicine (ZLM), Inselspital, Bern University Hospital, Bern, Switzerland; 6 Department of Hematology and Bone Marrow Transplantation, Rambam Health Care Campus, Haifa, Israel; 7 Department of Hematology, Shaare Zedek Medical Center, Jerusalem, Israel; 8 Division of Hematology, University Hospital Basel, Basel, Switzerland

**Keywords:** Stem-cell research, Haematopoietic stem cells

## Abstract

Autologous hematopoietic cell transplantation (HCT) is suitable for consolidation of favorable-/intermediate-risk AML patients in CR1. However, ~50% of AML patients relapse after autologous HCT, and efficacy of subsequent salvage strategies including allogeneic HCT remains unclear. We studied 123 consecutive patients with newly diagnosed AML undergoing high-dose chemotherapy (HDCT)/autologous HCT in CR1. In relapsing patients afterwards, we analyzed salvage treatments and outcomes focusing particularly on salvage allogeneic HCT. Of 123 patients, 64 (52%) relapsed after autologous HCT. Subsequently, 13 (21%) received palliative therapy, whereas 51 (79%) proceeded to salvage therapy with a curative intent. Of the 47 patients with a curative intent and who did not proceed directly to allogeneic HCT, 23 (49%) achieved CR2 or had ongoing hematologic CR1 despite molecular relapse. Finally, 30 patients (47%) received allogeneic HCT with estimated 3-year leukemia-free and overall survival rates of 33% and 43%. Hematologic remission at allogeneic HCT and lack of acute GvHD had a positive impact on OS and LFS (*p* < 0.05). Our study suggests that almost 80% of AML patients can undergo salvage therapy following relapse after front-line HDCT/autologous HCT. Allogeneic HCT can provide cure in one third of patients relapsing after front-line HDCT/autologous HCT.

## Introduction

Although up to 80% of AML patients less than 60 years of age achieve complete hematological remission (CR1) following intensive induction chemotherapy [1], more than half of them will ultimately relapse [2]. Consolidation of CR1 with high-dose chemotherapy (HDCT) followed by autologous hematopoietic cell transplantation (HCT) is one option in patients with favorable- or intermediate-risk AML—particularly in patients with minimal residual disease (MRD) negativity after intensive induction therapy. Various studies have demonstrated that autologous HCT performed in CR1 has a low non-relapse mortality (NRM) and is associated with a lower relapse rate than consolidation chemotherapy alone [3–7]. Durable responses following autologous HCT have been reported within distinct AML subtypes including core binding factor or *NPM1*-mutated AML [8, 9]. Allogeneic HCT is the preferred option in patients with adverse risk profiles, as the graft-versus-leukemia (GvL) effect [10, 11] cannot be provided by HDCT/autologous HCT outweighing in these patients the risks of graft-versus-host disease (GvHD).

Nevertheless, relapse remains the major problem for patients with AML. This is also true for patients after HDCT/autologous HCT, with relapse rates up to 50% [12]. MRD testing is increasingly performed in patients with a higher risk of post-autologous HCT relapse, e.g., due to MRD positivity following induction therapy [13, 14], and has triggered the interest in maintenance strategies post-transplant. Hypomethylating agents or (in case of the respective mutation) FLT3 inhibitors have demonstrated promising results for patients with an increased relapse risk after allogeneic HCT [15–18], whereas these options still await exploration in patients after autologous HCT.

Reluctant use of autologous HCT for consolidation of CR1 in AML is driven by concerns of the lacking GvL effect, the possibility of autologous graft contamination by leukemic stem cells, and the toxicity of the HDCT. Moreover, the preferred re-induction strategies in the relapse situation after autologous HCT are a matter of continuous debate, especially regarding the feasibility of subsequent salvage allogeneic HCT and considering the toxicity of the previous HDCT/autologous HCT [19–21]. To further explore feasibility and safety of such an approach, we here analyzed 123 consecutive AML patients consolidated with HDCT/autologous HCT in CR1. Particularly, we investigated strategies applied to treat relapse after autologous HCT consolidation in CR1 with a special focus on feasibility and outcome of allogeneic HCT as a salvage procedure in this scenario.

## Materials and methods

### Patients

This retrospective single-center study included 123 consecutive adult patients (≥18 years) diagnosed with AML and undergoing HDCT/autologous HCT in CR1 between 2000 and 2018 at the Department of Medical Oncology, University Hospital of Bern, Switzerland. Inclusion criteria were: (A) all types of AML including *de novo*, secondary (s-AML) following myelodysplastic syndrome, therapy-associated (t-AML), and extramedullary manifestation (chloroma), as well as biphenotypic leukemia. (B) Patients must have received two cycles of anthracycline/cytarabine-based induction therapy followed by consolidation by HDCT/autologous HCT. (C) The rationale for HDCT/autologous HCT was the achievement of MRD-negative CR1 by flow cytometry and molecular genetics (if appropriate markers were available) in AML patients with favorable of intermediate-risk profile with adequate performance status. Only occasionally, patients with adverse risk who either lacked a suitable donor or refused allogeneic HCT in CR1 were also included. (D) CD34+ stem cell mobilization with granulocyte colony-stimulating factor and stem cell harvest were performed after the second induction cycle [22, 23]. HDCT comprised busulfan (total dose 16 mg/kg p.o.) and cyclophosphamide (total 120 mg/kg i.v.) [24, 25].

Patients with evidence of relapse (morphological, cytogenetic, or molecular) occurring after consolidation by HDCT/autologous HCT in CR1 were subsequently analyzed. All allogeneic HCTs were performed at the Department of Hematology, University Hospital of Basel, Switzerland. European LeukemiaNet (ELN, 2017) criteria were used for genetic risk stratification of patients [26]. The study was approved by the local ethic committee (Decision #221/15) and conducted in compliance with the Declaration of Helsinki. The informed consent was obtained from all subjects.

### Endpoints

This retrospective analysis aimed to study relapse after autologous HCT consolidation in CR1 with a special focus on feasibility and outcome of allogeneic HCT as a salvage procedure. The following endpoints were considered: (A) cumulative incidence of relapse in patients with consolidation by HDCT/autologous HCT in CR1; (B) frequency of relapsing patients effectively receiving allogeneic HCT, interval from first relapse to allogeneic HCT, and NRM following allogeneic HCT; and (C) overall survival (OS) and leukemia-free survival (LFS) in patients with salvage allogeneic HCT after autologous HCT in CR1.

### Definitions

Staging and morphologic response criteria in AML were based on the 2017 ELN AML recommendations [26]. Molecular response criteria were used according to the consensus document from the European LeukemiaNet MRD Working Party [27]. OS was defined as time from allogeneic HCT to death from any cause. LFS was defined as time from allogeneic HCT to relapse or progression or death from any cause. NRM was defined as death without evidence of relapse or progression.

For allogeneic HCT, myeloablative conditioning (MAC) was defined as a regimen containing either a total dose of greater than 6.4 mg/kg busulfan i.v. or two alkylating agents. Regimens containing lower conditioning intensities were defined as reduced-intensity conditioning (RIC) [28]. Acute and chronic GvHD were categorized following international criteria [29, 30].

### Statistics

Categorical variables were summarized as frequencies and percentages, and continuous variables were summarized as median and range. Probabilities of OS and LFS were calculated using the Kaplan–Meier method. All analyses were performed using Statistical Software for Social Sciences version 26.0 (SPSS, Chicago, IL) and R Software for Statistical Computing and Graphics (R version 3.6.2).

## Results

### Characteristics of patients undergoing consolidation by HDCT/autologous HCT in CR1

This study included 123 consecutive AML patients undergoing intensive induction therapy with subsequent consolidation by HDCT/autologous HCT in CR1. We summarized patient and disease characteristics at diagnosis in Table 1. The median age at diagnosis was 54 (range, 19–71) years, and gender distribution was comparable (61 males: 62 females). *De novo* AML was the most common type (80%, 98/123) followed by s-AML (10%, 12/123), t-AML (3%, 4/123), and extramedullary manifestation/chloroma (2%, 3/123). Six (5%) patients had biphenotypic acute leukemia. A total of 102 (83%) patients had either favorable or intermediate-risk AML (41% and 42%, respectively), whereas 21 patients (17%) were considered as adverse risk.

**Table 1:** Characteristics of patients and disease at first diagnosis of the total cohort and as well specified for the patients who developed a relapse versus those who remained in continuous CR following front-line autologous HCT.

Parameter	Total cohort	Patients with relapse following autologous HCT	Patients in continuous CR following autologous HCT
*Patients with AML, number*	123	64	59
*Demographic characteristics*			
Males/females (ratio)	61/62 (0.98)	31/33 (0.93)	30/29 (1.03)
Median age, years (range)	54 (19–71)	54 (19–71)	58 (22–71)
*Type of AML*			
*De novo* AML, *n* (%)	98 (80%)	49 (77%)	49 (83%)
Secondary AML, *n* (%)	12 (10%)	10 (15%)	2 (3%)
Therapy-related AML, *n* (%)	4 (3%)	–	4 (7%)
Extramedullary manifestation (chloroma), *n* (%)	3 (2%)	2 (3%)	1 (2%)
Biphenotypic acute leukemia, *n* (%)	6 (5%)	3 (5%)	3 (5%)
*FAB subtypes*			
M0, *n* (%)	21 (17%)	18 (28%)	3 (5%)
M1, *n* (%)	23 (19%)	10 (15%)	13 (22%)
M2, *n* (%)	47 (38%)	20 (31%)	27 (46%)
M3, *n* (%)	1 (1%)	1 (2%)	–
M4, *n* (%)	18 (14%)	11 (17%)	7 (12%)
M5, *n* (%)	11 (9%)	3 (5%)	8 (13%)
M6, *n* (%)	2 (2%)	1 (2%)	1 (2%)
*Genetic risk groups (ELN 2017)*			
Favorable, *n* (%)	50 (41%)	20 (31%)	30 (51%)
Intermediate, *n* (%)	52 (42%)	28 (44%)	24 (41%)
Adverse, *n* (%)	21 (17%)	16 (25%)	5 (8%)
*Karyotype*			
Normal karyotype	73 (59%)	43 (67%)	30 (51%)
t(8;21)(q22;q22.1); *RUNX1-RUNX1T1*	12 (10%)	5 (8%)	7 (12%)
inv(16)(p13.1q22) or t(16;16)(p13.1;q22);*CBFB-MYH11*	6 (5%)	2 (3%)	4 (7%)
t(15;17)(q22;q21); *PML-RARA*	1 (1%)	1 (2%)	–
Complex karyotype (≥3 clonal aberrations)	13 (11%)	7 (11%)	6 (10%)
Others	18 (14%)	6 (9%)	12 (20%)
*Molecular techniques at diagnosis*			
PCR only	117 (95%)	58 (91%)	59 (98%)
PCR and NGS	6 (5%)	6 (9%)	–
*Mutation frequency*			
Patients positive for at least one mutation	82 (67%)	38 (59%)	44 (75%)
Patients without any mutations	41 (33%)	26 (41%)	15 (25%)
*Mutations (selection)*			
*NPM1*	34 (28%)	20 (31%)	14 (24%)
*FLT3*-ITD, total number	22 (18%)	12 (19%)	10 (17%)
*FLT3*-ITD as isolated mutation	10 (8%)	5 (8%)	5 (8%)
*FLT3*-ITD with mutated *NPM1*	12 (10%)	7 (11%)	5 (8%)
*CEBPA*	6 (5%)	1 (2%)	5 (8%)

*CR* complete remission, *HCT* hematopoietic stem cell transplantation, *AML* acute myeloid leukemia, *FAB* French–American–British classification of AML, *ELN* European LeukemiaNet, *PCR* polymerase chain reaction, *NGS*, next-generation sequencing.

### Relapsing patients following consolidation by HDCT/autologous HCT in CR1

**Fig. 1:**
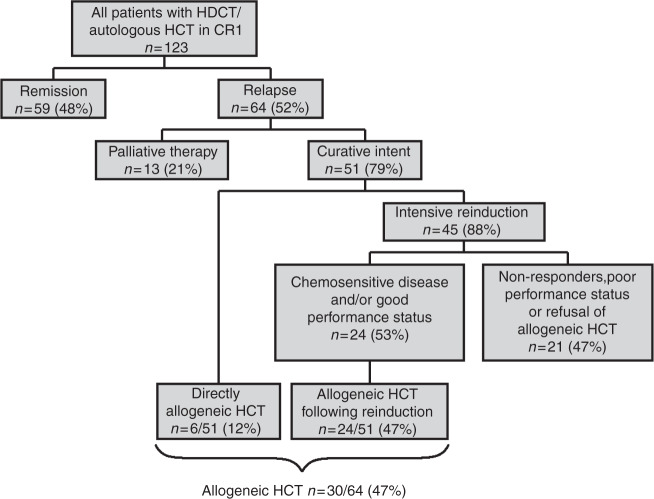
Consort diagram with number of patients undergoing HDCT/autologous HCT in CR1, remission status post autologous HCT, and treatment intentions following relapse post autologous HCT. HDCT high-dose chemotherapy, HCT hematopoietic cell transplantation, CR1 first complete remission.

Remission status following consolidation by HDCT/autologous HCT in CR1 and eventual relapse treatment are presented in Supplementary Table 1. Out of 123 patients, 64 (52%) patients relapsed after a median interval of six months after autologous HCT. Within the relapse group, 79% (51/64) patients underwent subsequent treatment with curative intent, whereas the remaining 21% (13/64) received palliative treatment regimens (Fig. 1 and Supplementary Table 1).

In total, 23 of 47 patients (49%) receiving salvage treatment with a curative intent at relapse (and who did not directly undergo allogeneic HCT) achieved CR2 or had ongoing hematologic CR1 under bridging therapy despite molecular relapse.

### Relapsing patients undergoing salvage allogeneic HCT

Characteristics of allogeneic HCT are presented in Table 2. In total, 30 out of 64 (47%) patients relapsing post HDCT/autologous HCT ultimately received allogeneic HCT, either directly/after non-intensive bridging (*n* = 6/30) or undergoing preceding intensive relapse treatment (*n* = 24/30) (Fig. 1). The median interval between autologous HCT and allogeneic HCT was 9 months, and the median interval between relapse after autologous HCT and allogeneic HCT was 2.5 months. Despite preceding HDCT/autologous HCT in CR1, most patients (77%, 23/30) were treated with MAC regimen, whereas only few patients (23%, 7/30) received RIC preceding allogeneic HCT.

**Table 2:** Therapy regimens and clinical outcome among 30 relapsed AML patients undergoing salvage therapy and subsequent allogeneic hematopoietic cell transplantation.

Parameter	*N* (%)
*Allogeneic HCT*	30
*Myeloablative conditioning*	23 (77)
BU/CY	15 (50)
CY/TBI	6 (21)
TBF-PTCy	1 (3)
EtoCy	1 (3)
*Reduced-intensity conditioning*	7 (23)
FLU/BU2	3 (10)
FLU/sTBI	2 (7)
FLAMSA	1 (3)
FluCyTBI-PTCy	1 (3)
*Donor type*	30
Matched related	11 (37)
Matched unrelated	11 (37)
Mismatched unrelated	4 (12)
Haploidentical	2 (7)
Umbilical cord blood	2 (7)
*Median interval from first relapse to allogeneic HCT (months, range)*	2.5 (1–9)
*Remission status at the time of allogeneic HCT*	30
CR	25 (83)
PR	1 (3)
Refractory disease	4 (14)
*GvHD frequency*	
aGvHD	8 (27)
cGvHD	11 (37)
*Remission status during follow-up after allogeneic HCT*	
Ongoing CR	19 (63)
Relapse/progression after allogeneic transplant	11 (37)
Hematological relapse	7
Molecular relapse only	3
CNS relapse	1
*Median time from autologous HCT to allogeneic HCT (months, range)*	9 (3–61)
*Median time to relapse following allogeneic HCT (months, range)*	6 (1–23)
*Relapse treatment following r/r disease after allogeneic HCT*	11
Azacitidine+/– DLIs	3/11 (28)
Sorafenib	3/11 (28)
ARA-C/Idarubicin	1/11 (8)
Whole brain radiotherapy followed by dasatinib maintenance	1/11 (8)
BSC	3/11 (28)
*Outcome of treatment following relapse after allogeneic HCT*	8/11
Refractory disease	6 (74.0)
Molecular remission	1 (13)
CNS remission	1 (13)
*Median interval from allogeneic HCT to last follow-up (months, range)*	28 (1–150)
*Survival status at last follow-up*	30
Alive and in remission	11 (37)
Death in CR due to NRM (9 GvHD, 1 infection)	10 (33)
Death due to progressing disease	9 (30)

*HCT* hematopoietic cell transplantation, *AML* acute myeloid leukemia, *BU/CY* busulfan, cyclophosphamide, *CY/TBI* cyclophosphamide, total body irradiation, *TBF-PTCy* thiothepa, busulfan, fludarabine, post-transplant cyclophosphamide, *EtoCy* etoposide, cyclophosphamide, *FLU/BU2* fludarabine, busulfan, *FLU/sTBI* fludarabine, single dose total body irradiation, *FLAMSA* fludarabine, amsacrine, cytarabine, *FluCyTBI-PTCy* fludarabine, cyclophosphamide, total body irradiation, post-transplant cyclophosphamide, *CR* complete remission, *PR* partial remission, *GvHD* graft-versus-host disease, *aGvHD* acute GvHD, *cGvHD* chronic GvHD, *CNS* central nervous system, *r/r* relapsed/refractory, *DLI* donor lymphocyte infusion, *ARA-C* cytarabine, *BSC* best supportive care, *NRM* non-relapse mortality.

Out of 30 patients, 22 (74%) received grafts from HLA-identical donors, equally distributed between related and unrelated donors (11 each). Eight patients (26%) had either mismatched (4/8), haploidentical (2/8) donors, or umbilical cord blood (2/8) as hematopoietic stem cell source. The remission status at the time of allogeneic HCT was as follows: 83% (25/30) were in CR2 or in CR1 with molecular relapse, 3% (1/30) had PR, and 14% (4/30) had refractory disease.

**Fig. 2:**
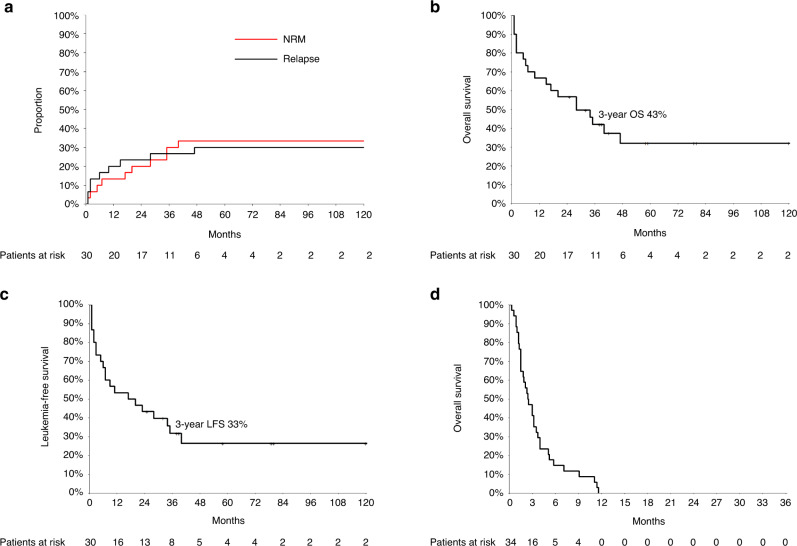
Outcomes of salvage allogeneic HCT and non-allogeneic HCT treatment in AML patients following relapse after front-line autologous HCT. **a** Cumulative incidence of relapse and non-relapse mortality in the salvage allogeneic HCT group following front-line autologous HCT. **b** Overall survival in the salvage allogeneic HCT group following relapse after front-line autologous HCT. **c** Leukemia-free survival in the salvage allogeneic HCT group following relapse after front-line autologous HCT. **d** Overall survival in the non-allogeneic HCT group following relapse after front-line autologous HCT.

The median interval since allogeneic HCT to last follow-up (FU) was 28 months. At last FU, 11 patients were alive (37% of the allogeneic HCT recipients) and in remission. Of these 11 patients with remission post-allogeneic HCT, 5 patients underwent conditioning treatment directly (4/5) or had bridging treatment with enasidenib (1/5) due to molecular relapse. Eleven patients (37%) suffered from post-transplant relapse or progression with a median time of 6 months following allogeneic HCT. Mortality was due either to non-relapse reasons (10/30; 33%) or relapsed/refractory disease (9/30; 30%) (Fig. 2a). Mortality in all non-relapse patients was due to infectious complications associated predominantly with GvHD (9/10) in post-allogeneic follow-up. Accordingly, the estimated 3-year OS and LFS were 43% and 33%, respectively, in the salvage allogeneic HCT group (Fig. 2b, c). Acute GvHD grade I–IV was observed in 27% (8/30) of the allogeneic HCT recipients, with five patients (17%) having grade III–IV. Eleven patients (37%) developed chronic GvHD.

Applying univariate risk factor analysis (Supplementary Table 2), gender, remission status at allogeneic HCT, and acute GvHD turned out to have a significant impact on OS and/or LFS. We observed that female gender contributed to worse OS (*p* = 0.032) and presence of severe acute GvHD (grade II–IV) negatively influenced both OS (*p* = 0.006) and LFS (*p* = 0.025). In contrast, complete hematologic remission at allogeneic HCT contributed to better LFS (*p* = 0.036) and OS (*p* = 0.011). None of the other risk factors (pretreatment characteristics or details at allogeneic transplantation) had a significant impact on OS and LFS.

### Patients without subsequent allogeneic HCT at relapse of AML after consolidation by HDCT/autologous HCT in CR1

Therapy regimens and the clinical outcomes for the 34 patients without subsequent allogeneic HCT (53%, 34/64) following relapse treatment after consolidation by HDCT/autologous HCT in CR1 are depicted in Supplementary Table 3. The reasons for not proceeding to allogeneic HCT in this group were as follows: 56% (19/34) due to refractory disease, 38% (13/34) due to poor general condition, and 6% (2/34) refused allogeneic HCT. The median survival from relapse was 2.5 months. At last FU, all 34 patients in this cohort had succumbed to progressive disease (Fig. 2d).

### Clinical outcomes in patients with stable remission following consolidation by HDCT/autologous HCT in CR1

In patients without relapse after consolidation by HDCT/autologous HCT in CR1, 50 out of 59 (85%) were alive in CR of AML with a median FU of 7 years. Of the remaining 9 patients (15%), 4 patients (7%) died from transplant-related mortality due to infection in aplasia in the early post-autologous HCT phase, 3 patients (5%) died due to co-morbidities (epileptic seizure, hernial incarceration, or unknown), and 2 (3%) due to a secondary malignancy occurring after autologous HCT.

## Discussion

HDCT followed by autologous HCT represents a suitable therapeutic option for consolidation of CR1 in good and intermediate-risk AML patients [4, 24, 31–33]. Among them, patients with negative MRD status following induction therapy particularly benefit from HDCT/autologous HCT treatment [7, 31, 34]. Acknowledging recent advances in AML treatment, prognosis of AML patients at relapse remains an unmet medical need with median survival rates of usually less than 1 year. Accordingly, treatment of patients relapsing after front-line autologous HCT remains challenging. In addition, concerns exist about tolerability of intensive chemotherapy and subsequent salvage allogeneic HCT in patients after preceding HDCT/autologous HCT. In the present study, we have analyzed the outcomes of salvage regimens with a focus on feasibility and efficacy of allogeneic HCT in a cohort of 123 consecutive AML patients who had received consolidation by HDCT/autologous HCT in CR1. The aim of our study was to evaluate the impact of preceding front-line HDCT/autologous HCT on the results of subsequent salvage therapies including allogeneic HCT in patients relapsing after HDCT/autologous HCT.

First, the relapse rate after consolidation with HDCT/autologous HCT in CR1 (52%) within our patient cohort was in accordance with previous data on autologous HCT-based consolidation in AML [9, 12, 24, 31, 35]. Of note, 17% of all patients at first diagnosis presented adverse cytogenetics and 18% fell into otherwise unfavorable AML categories (such as s-AML) being initially susceptible to increased risk for relapse post HDCT/autologous HCT [36]. In our cohort, 79% of patients relapsing after autologous HCT received salvage therapy with a curative intent, and most of them received intensive re-induction therapy. This suggests that HDCT/autologous HCT did not impact significantly on the eligibility for intensive salvage therapies.

Forty-nine percent of patients relapsing after first-line HDCT/autologous HCT and undergoing salvage re-induction were achieving CR2 in our cohort. This was similar to the CR2 rates reported by others for AML patients relapsing after conventional chemotherapy and receiving salvage chemotherapy treatment [37, 38]. In addition, 47% of all patients relapsing after HDCT/autologous HCT ultimately proceeded to salvage allogeneic HCT, which seems comparable to the percentage of patients undergoing intensive re-induction following relapse after conventional chemotherapy consolidation of CR1 [39]. Considering these results, we found no evidence that preceding HDCT/autologous HCT affects the proportion of relapsing AML patients admitted to salvage allogeneic HCT. In fact, MAC could be applied to the majority of patients (77%) post autologous HCT.

Importantly, the 3-year OS rate in the salvage-allo-cohort was 43%, which is comparable to reports in the literature in patients undergoing salvage allogeneic HCT with preceding chemotherapy consolidation of CR1 [40]. In all, 37% of patients in the salvage allogeneic HCT group relapsed resulting in a 3-year LFS of 33%. Again, this was consistent with the data reported by others for salvage allogeneic HCT in AML patients transplanted beyond CR1 with preceding conventional chemotherapy only [40]. In univariate risk factor analysis for survival outcomes, hematologic remission at allogeneic HCT and lack of severe acute GvHD had a positive impact on OS and LFS.

In comparison to front-line allogeneic HCT, salvage allogeneic HCT is known to be associated with higher NRM up to 46% in poor risk AML patients in the post-transplant FU [40–43]. Accordingly, in our study, the NRM in the salvage-allo-cohort was 33% and predominantly triggered by infectious complications associated with GvHD. Of note, no patient in our cohort had hepatic veno-occlusive disease as a result of liver injury due to the preceding HDCT. Recently, Christopeit et al. retrospectively summarized EBMT results of salvage allogeneic HCT for 537 patients in CR2 or at first hematologic relapse after consolidation by HDCT/autologous HCT in CR1. At 3 years post allograft, OS was 39.5%, LFS 31.4%, relapse incidence 34.6%, and NRM 33.7% [19]; and our results appear in accordance with this large multicenter registry study. In contrast, Foran et al. reported a CIBMTR cohort with long-term OS and LFS of 22% and 20%, respectively, for 302 patients undergoing salvage allogeneic HCT following treatment failure after consolidation by HDCT/autologous HCT in CR1, whereas this cohort was treated between 1995 and 2005 suggesting a possible time bias and recent improvements in treatment modalities [21].

Improvement and integration of NGS technologies in the diagnostic work-up have enabled to depict a unique genetic make-up for each individual AML case. In some situations, a molecular relapse may allow timely planning of allogeneic HCT, even before it becomes apparent at the morphological level [44–46]. This, on the one hand, results in fewer patients receiving autologous HCT while still being MRD-positive, whereas it may also allow more patients relapsing after autologous HCT with low leukemia burden to directly undergo salvage allogeneic HCT. Of note, all five patients in our cohort who underwent salvage allogeneic HCT directly following molecular relapse still remain in morphologic remission and are alive at last FU.

In conclusion, our study demonstrates that consolidation by HDCT/autologous HCT in CR1 is not negatively affecting feasibility and efficacy of subsequent allogeneic HCT for those patients relapsing after autologous HCT, and more than one third of patients relapsing after consolidation by HDCT/autologous HCT in CR1 can be rescued by salvage allogeneic HCT. As there is no curative alternative to allogeneic HCT in relapsing AML patients, the indication to salvage allogeneic HCT must be considered by treating physicians for patients relapsing after consolidation of CR1 with HDCT/autologous HCT in patients who are fit for this approach.

## Supplementary information

Supplementary Tables 1–3
