# Supplementary material for: Feasibility and efficacy of salvage allogeneic stem cell transplantation in AML patients relapsing after autologous stem cell transplantation
Source: Bone Marrow Transplant. 2021 Nov 13;57(2):224–31. doi: 10.1038/s41409-021-01521-5 (PMC8821015; doi:10.1038/s41409-021-01521-5)
Supplement: Supplementary file 1 — Supplementary Tables 1–3 [file 41409_2021_1521_MOESM1_ESM.docx]

**Supplemental Table 1.** Remission status following front-line autologous HCT and relapse therapy among patients of this study.

| **Therapies** | **№ of patients** |
| --- | --- |
| **Study cohort** | **123** |
| **Induction** | |
| Anthracycline/ARA-C based regimens (2 cycles), n (%) | 123 (100%) |
| Median interval from diagnosis to HDCT/autologous HCT (weeks, range) | 15 (10-45) |
| **Maintenance therapy following front-line HDCT/autologous HCT**, n (%) | 6/123 (5%) |
| Sorafenib, n (%) | 4 (3%) |
| Imatinib, n (%) | 2 (2%) |
| **Relapse after front-line HDCT/autologous HCT**, n (%) | 64 (52%) |
| **Type of relapse after front-line HDCT/autologous HCT** | 64 |
| Hematologic, n (%) | 53 (83%) |
| Cytogenetic only, n (%) | 1 (1%) |
| Molecular only, n (%) | 10 (16%) |
| **Median interval from first diagnosis to relapse, months (range)** | 10 (4-60) |
| Median interval from HDCT/autologous HCT to relapse, months (range) | 6 (0.5-57) |
| **Treatment of relapse after autologous HCT** | 64 |
| Intensive re-induction therapy, n (%) | 45 (70%) |
| CLAG- or FLAG-Ida based, n (%) | 25 (39%) |
| HAM, n (%) | 16 (25%) |
| ARA-C based 7+3 regimen, n (%) | 3 (5%) |
| Melphalan directly followed by 2nd autologous HCT, n (%) | 1 (1%) |
| Non-intensive bridging therapy (azacitidine/enasidenib) to allogeneic HCT, n (%) | 2 (1/1) (3%) |
| Directly myeloablative conditioning followed by allogeneic HCT, n (%) | 4 (6%) |
| Palliative therapy, n (%) | 13 (21%) |
| Palliative relapse therapy, n (%) | 8 (13%) |
| BSC, n (%) | 5 (8%) |
| **Response rate following intensive re-induction or non-intensive bridging therapy** | **47** |
| CR, n (%) | 23 (49%) |
| PR, n (%) | 1 (2%) |
| Refractory disease, n (%) | 23 (49%) |
| **Response rate following palliative relapse therapy** | **8** |
| Refractory disease, n (%) | 6 (75%) |
| Temporary cytoreduction, n (%) | 2 (25%) |
| **Consolidation of salvage treatment with allogeneic HCT, n (%)** | **30 (47%)** |
| **Therapies** | **№ of patients** |
| **Study cohort** | **123** |
| **Induction** | |
| Anthracycline/ARA-C based regimens (2 cycles), n (%) | 123 (100%) |
| Median interval from diagnosis to HDCT/autologous HCT (weeks, range) | 15 (10-45) |
| **Maintenance therapy following front-line HDCT/autologous HCT**, n (%) | 6/123 (5%) |
| Sorafenib, n (%) | 4 (3%) |
| Imatinib, n (%) | 2 (2%) |
| **Relapse after front-line HDCT/autologous HCT**, n (%) | 64 (52%) |
| **Type of relapse after front-line HDCT/autologous HCT** | 64 |
| Hematologic, n (%) | 53 (83%) |
| Cytogenetic only, n (%) | 1 (1%) |
| Molecular only, n (%) | 10 (16%) |
| **Median interval from first diagnosis to relapse, months (range)** | 10 (4-60) |
| Median interval from HDCT/autologous HCT to relapse, months (range) | 6 (0.5-57) |
| **Treatment of relapse after autologous HCT** | 64 |
| Intensive re-induction therapy, n (%) | 45 (70%) |
| CLAG- or FLAG-Ida based, n (%) | 25 (39%) |
| HAM, n (%) | 16 (25%) |
| ARA-C based 7+3 regimen, n (%) | 3 (5%) |
| Melphalan directly followed by 2nd autologous HCT, n (%) | 1 (1%) |
| Non-intensive bridging therapy (azacitidine/enasidenib) to allogeneic HCT, n (%) | 2 (1/1) (3%) |
| Directly myeloablative conditioning followed by allogeneic HCT, n (%) | 4 (6%) |
| Palliative therapy, n (%) | 13 (21%) |
| Palliative relapse therapy, n (%) | 8 (13%) |
| BSC, n (%) | 5 (8%) |
| **Response rate following intensive re-induction or non-intensive bridging therapy** | **47** |
| CR, n (%) | 23 (49%) |
| PR, n (%) | 1 (2%) |
| Refractory disease, n (%) | 23 (49%) |
| **Response rate following palliative relapse therapy** | **8** |
| Refractory disease, n (%) | 6 (75%) |
| Temporary cytoreduction, n (%) | 2 (25%) |
| **Consolidation of salvage treatment with allogeneic HCT, n (%)** | **30 (47%)** |

HCT, hematopoietic cell transplantation; ARA-C, cytarabine; HDCT, high-dose chemotherapy; CLAG-Ida, cladribine, cytarabine, granulocyte stimulating factor (G-CSF), idarubicin; FLAG-Ida, fludarabine, cytarabine, G-CSF, idarubicin; HAM, high-dose cytarabine, mitoxantrone; BSC, best supportive care; CR, complete remission; PR, partial remission.

**Supplemental Table 2.** Univariate risk factor analysis for LFS and OS in AML patients undergoing salvage allogeneic HCT after preceding front-line HDCT/autologous HCT.

| **Variable** | **Median LFS for all patients** | | **Median OS for all patients** | |
| --- | --- | --- | --- | --- |
|  | Months (95% CI) | *P*-value | Months (95% CI) | *P*-value |
| **Gender** | | | | |
| Male | (0-50) | 0.132 | - | **0.032** |
| Female | (0-13) |  | (0-34) |  |
| **Age at salvage allogeneic HCT** | | | | |
| ≤54 years (median) | (0-29) | 0.609 | (0-41) | 0.287 |
| >54 years | (0-42) |  | (12-58) |  |
| **Duration of CR1 after autologous HCT** | | | | |
| ≤7 months (median) | (0-28) | 0.225 | (0-63) | 0.261 |
| >7 months | (0-57) |  | (11-58) |  |
| **Time from autologous to salvage allogeneic HCT** | | | | |
| ≤9 months (median) | (8-49) | 0.094 | (0-62) | 0.153 |
| >9 months | (0-36) |  | (9-61) |  |
| **Remission status at salvage allogeneic HCT** | | | | |
| CR | (6-40) | **0.036** | (20-52) | **0.011** |
| Non-CR | (0.8-1.2) |  | (0.8-1.2) |  |
| **Donor type for salvage allogeneic HCT** | | | | |
| Matched donor | (0-61) | 0.054 | (18-63) | 0.195 |
| Non-matched donor* | (2-11) |  | (2-29) |  |
| **Conditioning regimen before salvage allogeneic HCT** | | | | |
| RIC | (0-29) | 0.953 | - | 0.705 |
| MAC | (0-45) |  | (8-48) |  |
| **Acute GvHD (grade II-IV) after allogeneic HCT** | | | | |
| No | (6-51) | **0.025** | - | **0.006** |
| Yes | (0-7) |  | (0-20) |  |
| **Chronic GvHD after allogeneic HCT** | | | | |
| No | (0-43) | 0.746 | (0-69) | 0.982 |
| Yes | (2-33) |  | (3-54) |  |

LFS, leukemia-free survival; OS, overall survival; AML, acute myeloid leukemia; HCT, hematopoietic cell transplantation; HDCT, high-dose chemotherapy; CR, hematologic remission; CR1, 1^st^ CR; RIC, reduced-intensity

conditioning; MAC, myeloablative conditioning; GvHD, graft-versus-host disease. *mismatched, haploidentical,

umbilical cord blood.

**Supplemental Table 3.** Therapy regimens and clinical outcomes among non-transplant patients following AML relapse after front-line HDCT/autologous HCT; clinical outcomes in the non-relapse group after front-line HDCT/autologous HCT.

| **Therapies** | **№ of patients** |
| --- | --- |
| **Patients without allogeneic HCT** | **34** |
| Median follow-up (starting from the frontline autologous HCT), months (range) | 7 (0.1-18.0) |
| **Relapse therapy - overview** |  |
| One relapse treatment line only, n (%) | 16 (47%) |
| Two relapse treatment lines, n (%) | 12 (35%) |
| Three relapse treatment lines, n (%) | 1 (3%) |
| BSC only, n (%) | 5 (15%) |
| **First-line relapse treatment in non-(allo) transplant group** | 29 |
| **Intensive re-induction**, n (%) | **21 (72%)** |
| - CLAG-/FLAG-Ida, n | 9 |
| - HAM, n | 11 |
| - ARA-C/Ida, n | 1 |
| **Non-intensive relapse therapy**, n (%) | **8 (28%)** |
| - Hydroxyurea, n | 3 |
| - Azacitidine, n | 3 |
| - Decitabine, n | 1 |
| - Sorafenib, n | 1 |
| **Second-line relapse treatment in the non-(allo) transplant group**, n (%) | **13 (45%)** |
| Intensive therapy, n | 2 |
| - FLAG-Ida, n | 1 |
| - ARA-C/Etoposide/Gemtuzumab Ozogamicin, n | 1 |
| Palliative chemotherapy/pharmacotherapy, n | 10 |
| - Hydroxyurea, n | 3 |
| - ARA-C, n | 2 |
| - Azacitidine, n | 2 |
| - Sorafenib, n | 2 |
| - Idarubicin only, n | 1 |
| **Third-line relapse treatment in the non-(allo) transplant group**, n (%) | **1 (3%)** |
| - Sorafenib, n | 1 |
| **Best response in the non-(allo/auto-)transplant group under relapse therapies, n** | **29** |
| CR, n (%) | 3 (10%) |
| SD, n (%) | 2 (7%) |
| Refractory disease, n (%) | 24 (83%) |
| **Median interval from relapse to last FU in the non-(allo) transplant group, months (range)** | **2.5 (0.3-11.3)** |
| **Remission/survival status at last FU in the non-(allo/auto-) transplant group** |  |
| Death from r/r disease, n (%) | 34 (100%) |
| **Survival status at last FU in the non-relapse group, n (%)** | **59 (100%)** |
| Alive, n (%) | 50 (85%) |
| - in CR, n (%) | 49 (83%) |
| - in CR, but with second malignancy, n (%) | 1 (2%) |
| Dead, n (%) | 9 (15%) |
| - Death in CR due to TRM, n (%) | 4 (7%) |
| - Death in CR due to co-morbidities, n (%) | 3 (5%) |
| - Death in CR due to second malignancy, n (%) | 2 (3%) |

AML, acute myeloid leukemia; HDCT, high-dose chemotherapy; HCT, hematopoietic cell transplantation; BSC, best supportive care; CLAG-Ida, cladribine, cytarabine, G-CSF, idarubicin; FLAG-Ida, fludarabine, cytarabine, G-CSF, idarubicin; HAM, high-dose cytarabine, mitoxantrone; ARA-C/Ida, cytarabine, idarubicin; CR, complete remission; SD, stable disease; FU, follow-up; TRM, transplant-related mortality.
